# Follicular traction urticaria*

**DOI:** 10.1590/abd1806-4841.20164654

**Published:** 2016

**Authors:** Hatice Duman, Ilteris Oguz Topal, Emek Kocaturk

**Affiliations:** 1 Okmeydani Training and Research Hospital – Istanbul, Turkey

**Keywords:** Urticaria, Traction, Follicular, Inducible

## Abstract

Inducible urticaria is a heterogeneous subgroup of chronic urticarias caused by a wide variety of environmental stimuli, such as exercise, cold, heat, pressure, sunlight, vibration, and water. A new term, follicular traction urticaria, was suggested as an unusual form of inducible urticarias. We report a patient who was diagnosed with follicular traction urticaria.

## INTRODUCTION

Inducible urticaria is a heterogeneous subgroup of chronic urticarias caused by a wide variety of environmental stimuli, such as exercise, cold, heat, pressure, sunlight, vibration, and water. The prevalence of inducible urticaria in the general population is approximately 0.5%.^1^ A new term, follicular traction urticaria, was suggested as an unusual form of inducible urticarias.^2,3^ We report a patient who was diagnosed with follicular traction urticaria.

## CASE REPORT

A 29-year-old woman presented to our outpatient clinic with a history of pruritic wheals. The lesions appeared on the skin as 2 mm to 5 mm wheals one year before. Urticarial papules appeared 5-15 minutes after waxing or using an electric epilator on the legs and forearms, but the reaction was spontaneously resolved within one to two hours. Eyebrow plucking with tweezers did not provoke lesions but caused erythema and mild pruritus. The patient reported no spontaneous urticaria attacks or other type of urticaria. No history of drug intake or systemic disease was reported.

**Figure 1:**
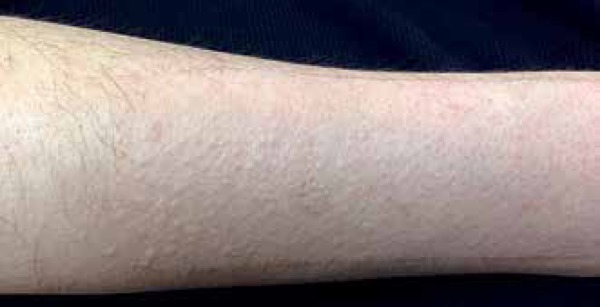
Follicular whealing precipitated by hair removal after waxing

**Figure 2:**
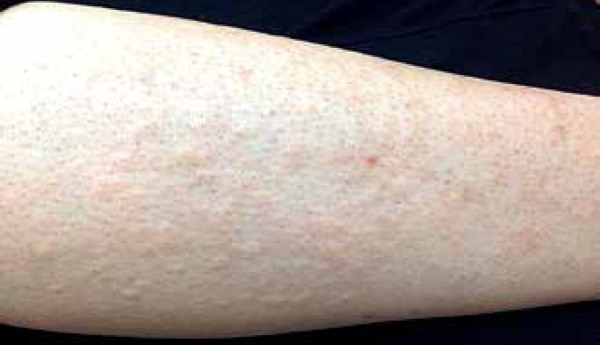
Follicular whealing precipitated by hair removal with electric epilator

Urticarial lesions could not be reproduced by stroking with a blunt object on the patient's flexor and extensor surface of the forearm and back, or by skin traction or gentle hair traction. Reddish follicular urticarial papules appeared on three tests: within 10 minutes on a hair removal test with wax on the right forearm; within 15 minutes on a hair removal test with an electric epilator on the leg; and within 20 minutes after a hair removal test with non-woven plaster on the left forearm (3x5 cm area) (Figures 1 and 2). Papules increased slowly and after 20–30 minutes showed regression. The reaction resolved completely within two hours.

Laboratory assessment revealed a normal complete blood count, erythrocyte sedimentation rate, C-reactive protein levels, antistreptolysin O levels, antibody titers (antithyroid, antinuclear, and rheumatoid factor), total immunoglobulin E (IgE), as well as renal, thyroid, and liver functions. Serological tests for hepatitis B and C were negative. Chest X-ray, throat culture, and stool examination for parasites were normal. Urinalysis was normal and urine culture was negative. Only the *Helicobacter pylori* stool antigen test was positive and eradication treatment was given. After the therapy, although the control *Helicobacter pylori* stool antigen was negative, the symptoms were not resolved. The reaction could be highly inhibited by 20 mg rupatadine 30–60 minutes before the hair removal and 20 mg rupatadine after the hair removal.

## DISCUSSION

Inducible urticaria is a heterogeneous subgroup of chronic urticaria, in which wheals are elicited by exogenous physical stimuli. Skin stroking, cold, heat, pressure, sunlight, water, exercise, and vibration are the common triggers.^1,4^ Two or more different subtypes of urticaria can coexist in the same patient.^1^

Gallo *et al.*^3^ has recently reported a rare form of inducible urticaria, traction urticaria, in which wheals were believed to be mostly triggered by skin traction. However, they could also reveal follicular wheals by gentle hair traction.^3^ Özkaya and Yazganoğlu reported a similar case of follicular traction urticaria.^2^ The present report agrees with Özkaya and Yazganoğlu's study revealing that hair traction was the triggering factor for the wheals. However, no urticarial lesions could be elicited by stroking the patient with a blunt object in our case. Based on a literature search, this is the second case of follicular traction urticaria reported in Turkey and the third case in the literature. Shelley and Shelley^5^ reported a case of follicular wheals caused by firm strokes with the broad edge of a tongue blade. The authors differentiated the lesions from follicular traction urticaria and labeled them as follicular dermatographism.

Shelley and Shelley^5^ revealed that firm strokes release the antigen from the bloodstream to trigger focal urticaria in sites with a high density of mast cells, namely around the hair follicle. The rationale was that the traction of hairs might release a different kind of antigen from the hair structure that interacts with IgE-sensitized mast cells around the hair follicle.

Follicular traction should also be considered among the triggers of inducible urticaria. Hair traction or skin stroking differentiates the type of the follicular whealing. We believe that when the number of patients with follicular traction urticaria increases, guidelines for a subtype of inducible urticaria shall be composed.
